# Editorial: Fruit and seed evolution in angiosperms

**DOI:** 10.3389/fpls.2023.1196443

**Published:** 2023-04-19

**Authors:** Alexander P. Sukhorukov, Mariane S. Sousa-Baena, Mikhail S. Romanov, Xin Wang

**Affiliations:** ^1^ Department of Higher Plants, Biological Faculty, M.V. Lomonosov Moscow State University, Moscow, Russia; ^2^ Laboratory Herbarium (TK), Tomsk State University, Tomsk, Russia; ^3^ School of Integrative Plant Science and L.H. Bailey Hortorium, Section of Plant Biology, Cornell University, Ithaca, NY, United States; ^4^ Laboratory of Tropical Plants, Tsitsin Main Botanical Garden of Russian Academy of Sciences, Moscow, Russia; ^5^ State Key Laboratory of Palaeobiology and Stratigraphy, Nanjing Institute of Geology and Palaeontology and CAS Center for Excellence in Life and Paleoenvironment, Chinese Academy of Sciences, Nanjing, China

**Keywords:** angiosperm, reproductive organ, fruit, seed, evolution, developmental anatomy, molecular phylogeny

## 1 Introduction

The diversity of fruits and seeds is enormous all over the world, and they play a crucial role in all aspects of biology, including human life. Despite the long history of carpological research, many characteristics of fruit and seed structure and evolution, as well as dispersal mechanisms, seed germination, and the ecological parameters of fruit and seed traits, have been lacking attention so far. Recently developed comparative methods based on molecular phylogeny and other new technologies provide an excellent opportunity for tracing the evolution of fruits and seeds in various plant groups.

The Research Topic is aimed at presenting recent discoveries concerning several aspects of fruit and seed traits as follows: (1) their importance for understanding taxonomic relationships, because reproductive characteristics have low phenotypic plasticity, thus being stable and suitable for classification purposes; (2) their evolutionary patterns in different taxa and the role they have played in the evolution of each examined taxon as a whole; and (3) the ecological consequences of their changes throughout evolution in different taxa. We collected original research articles and reviews from the following subject areas: (a) fruit structure and seed structure and their evolution in a phylogenetic context, (b) comparative carpological research on plants from different plant communities and ecological niches, (c) new insights into fruit and seed dispersal, and (d) recent discoveries about features of seed germination.

In total, five original research papers and one review were published. In their study, Song et al. evaluated the importance of seed characters in the large genus *Impatiens* L. (Balsaminaceae) from different points of view—the taxonomic significance of seed characters and their evolution in this genus—and discussed seed coat diversity with reference to dispersal and environmental adaptations.

The article by Sbais et al. is a deep investigation of seed coat anatomy and its evolution in the subtribe Eugeniinae (Myrtaceae). They showed that dehiscent fruits can be considered a plesiomorphic state in Myrtaceae, with an ancestor of the family having seeds featuring a completely sclerified testa. They concluded that the other testa types described for the current species with either dehiscent or indehiscent fruits are simplified versions of the ancestral type.

Another paper, by Sukhorukov et al. is focused on the anatomical features, evolutionary trends, and ecological significance of various morphoanatomical structures found in the seeds of Aizoaceae. All 18 discovered characteristics were compared with those of other families from core Caryophyllales. Reduction to one-seeded or synaptospermic fruits has been accompanied by drastic changes in the structure of these seeds and their mode of dispersal, as compared to close relatives with multi-seeded capsules. In many species, hygrochasy is combined with a thin seed coat, thereby playing a crucial part in the rapid germination of these seeds under semiarid conditions.

Using various techniques, Wang et al. associated floral thermogenesis in *Magnolia denudata* with the biosynthesis of volatile organic compounds (VOCs), the upregulation of alternative oxidase (AOX) and of genes modulating lignin catabolism, and the increased activity of AOX respiration. Their results revealed that AOX plays a central role in the coupling of thermogenesis with VOC biosynthesis during the anthesis of *Magnolia denudata*.

On the basis of the research into *LEAFY COTYLEDON1* (*LEC1*) gene functions in *Adiantum capillus-veneris*, in their review, Bai et al. advanced a “golden-trio hypothesis” to explain the origin of seeds. A “seed program” can be interpreted as the integration of three key events: assimilating flow, ABA-mediated stress responses, and stress-induced *LEC1* expression. A series of joint physiological and morphological innovations had led to the origin of seeds.

Paczesniak et al. characterized seed size variation, endosperm formation, and embryo viability in a comprehensive set of sexual and apomictic *Boechera* lineages. They demonstrated that seed mass is an important predictor of several traits, including germination probability and timing, with heavier seeds having higher probability of germination, germinating earlier, and possessing higher root growth rates. They showed that seed mass is under natural selection and provides specific advantages to apomictic embryos, because selection against small seeds is stronger in apomictic than in sexual embryos.

## 2 Prospects

Some key parameters of fruits and seeds are poorly studied and should receive more attention in the future, particularly seed adaptations to new ecological and climatic factors in the context of weed syndrome as well as the fruit and seed structure of plants playing different roles in natural plant communities, especially those of desert plant species. Additionally, the involvement of fruits and seeds in plant–animal interactions and the evolution and development of fruit and seed structure, though well studied in some taxa, still need to be addressed at broader phylogenetic and spatial scales.

## Author contributions

All authors contributed equally to the design, writing, and editing of the manuscript. All authors contributed to the article and approved the submitted version.

